# Anti-Axl antibody treatment reduces the severity of experimental autoimmune encephalomyelitis

**DOI:** 10.1186/s12974-020-01982-3

**Published:** 2020-10-29

**Authors:** Juwen C. DuBois, Alex K. Ray, Peter Davies, Bridget Shafit-Zagardo

**Affiliations:** 1 Department of Pathology, Albert Einstein College of Medicine, 1300 Morris Park Ave., Bronx, NY 10461 USA; 2 Department of Microbiology and Immunology, Albert Einstein College of Medicine, Bronx, NY USA; 3 North Shore-LIJ Health System, Feinstein Institute for Medical Research, Manhasset, NY USA

**Keywords:** α*-*Axl antibody, EAE, TAM activation, Demyelination, Microglia

## Abstract

**Background:**

Multiple sclerosis is an immune-mediated disease of the central nervous system (CNS) characterized by inflammation, oligodendrocytes loss, demyelination, and damaged axons. Tyro3, Axl, and MerTK belong to a family of receptor tyrosine kinases that regulate innate immune responses and CNS homeostasis. During experimental autoimmune encephalomyelitis (EAE), the mRNA expression of MerTK, Gas6, and Axl significantly increase, whereas Tyro3 and ProS1 remain unchanged. We have shown that Gas6 is neuroprotective during EAE, and since Gas6 activation of Axl may be necessary for conferring neuroprotection, we sought to determine whether α-Axl or α-MerTK antibodies, shown by others to activate their respective receptors in vivo, could effectively reduce inflammation and neurodegeneration.

**Methods:**

Mice received either α-Axl, α-MerTK, IgG isotype control, or PBS before the onset of EAE symptoms. EAE clinical course, axonal damage, demyelination, cytokine production, and immune cell activation in the CNS were used to determine the severity of EAE.

**Results:**

α-Axl antibody treatment significantly decreased the EAE clinical indices of female mice during chronic EAE and of male mice during both acute and chronic phases. The number of days mice were severely paralyzed also significantly decreased with α-Axl treatment. Inflammatory macrophages/microglia and the extent of demyelination significantly decreased in the spinal cords of α-Axl-treated mice during chronic EAE, with no differences in the production of pro-inflammatory cytokines. α-MerTK antibody did not influence EAE induction or progression.

**Conclusion:**

Our data suggests that the beneficial effect of Gas6/Axl signaling observed in mice administered with Gas6 can be partially preserved by administering an activating α-Axl antibody, but not α-MerTK.

## Background

Tyro3, Axl, and MerTK (TAM) are a family of receptor tyrosine kinases that are differentially activated by their ligand growth arrest-specific gene 6 (Gas6) and ProteinS1 (ProS1). Although both Gas6 and Pros1 share common features and require γ-carboxylation for their activity, they differ in specificity and affinity for Tyro3, Axl, and MerTK [1]. Gas6 is the sole ligand for Axl; however, higher concentrations are required to activate Tyro3 and MerTK, with a relative affinity as follows: Axl > Tyro3 > MerTK [2]. ProS1, another vitamin K-dependent circulating protein, preferentially binds to Tyro3 and MerTK and does not activate Axl [3, 4]. Gas6 is primarily expressed in the central nervous system (CNS) [5], in the cytosol of neurons and astrocytes [6], whereas Axl is widely expressed in neurons, oligodendrocytes, astrocytes, and microglia in the CNS and in the kidneys, liver, and heart [7–13]. Axl has low tissue specificity and is the most highly expressed skeletal muscle and testis (myocytes); however, Nowakowski et al. demonstrated that Axl is strongly expressed in the radial glia, brain capillaries, and microglia, and its expression is conserved in rodents and human cerebral model systems [11].

Gas6-Axl signaling is involved in the regulation of innate immune responses, cell growth and survival, mitogenesis, CNS homeostasis, and myelination [14–17], and several studies have demonstrated the benefits of Gas6 signaling in the CNS [16, 18–22]. We have previously shown that the addition of Gas6 to the brains of cuprizone-treated mice enhances remyelination, and when administered to myelin oligodendrocyte glycoprotein (MOG)-sensitized C57Bl/6J mice, it significantly improves the clinical outcome and recovery from MOG-induced experimental autoimmune encephalomyelitis (EAE) [22, 23]. However, the loss of Axl signaling in Axl^−/−^ mice results in enhanced inflammation in the CNS during MOG-induced EAE [24]. Although ProS1 shares 40% homology with Gas6 and can activate Tyro3 and MerTK, our previous work did not show any detectable increase in ProS1 expression in the CNS, suggesting that ProS1 cannot substitute for Gas6 [22]. Furthermore, since Axl is upregulated during EAE in WT and Tyro3^−/−^ mice, and Tyro3^−/−^ mice are not more severely affected than WT mice following MOG sensitization, this suggests that the protective effect of Gas6 is via Gas6/Axl signaling [24]. Additionally, loss of Gas6/Axl signaling results in less remyelination following cuprizone exposure, indicating that ProS1/Tyro3/MerTK signaling cannot compensate for the loss of Gas6 and Axl [18].

Our previous studies demonstrated that Axl^−/−^ and Axl^−/−^/Gas6^−/−^ mice have more lipid-laden/myelin debris relative to WT mice during cuprizone intoxication and increased demyelination during EAE, suggesting that Gas6/Axl signaling is significantly beneficial for reducing inflammation and clearance of dead cells and myelin debris in mice [18, 24, 25]. In multiple sclerosis lesions and in mouse models of demyelinating disease, dysregulation of the Gas6 receptor contributes to a poorer outcome and prolonged lesion activity [26, 27]. Therefore, we sought to characterize the role of Axl and MerTK in the preservation of CNS function and recovery following MOG_35–55_-induced EAE, a model of CNS inflammation that shares several clinical and pathologic features with multiple sclerosis (MS). Induction of EAE results in infiltration of T cells and monocytes, increased inflammation, expression of pro-inflammatory molecules, demyelination, and axonal damage. We hypothesized that the administration of an activating α-Axl but not MerTK antibody during EAE would decrease disease severity, enhance recovery, and reduce long-term axonal damage by protecting myelin.

The novelty of this study lies in the use of an antibody that has been shown to activate the Axl receptor in mice [28]. The authors showed that when the α-Axl antibody was injected intravenously, it was capable of activating the Axl receptor in vivo and in vitro [28]. Phosphorylated Tyro3 (p-Tyr) and soluble Axl (s-Axl) were used as a measure of activation, and they show that 10 μg of the α-Axl antibody maintained p-Tyr for at least 24 h and s-Axl for at least 7 days [28]. We targeted the Axl receptor using this Axl-activating antibody as a more focused approach to determine whether Axl-induced activation confers neuroprotection against axonal damage and loss, commonly observed during chronic MS. We also examined the effect of a MerTK-activating antibody. In activating Axl and MerTK during EAE using antibodies known to activate these receptors, we aimed to identify any therapeutic benefit resulting from changes in pro-inflammatory signaling molecules, myelination, and integrity of axons.

## Methods

### Animals

C57Bl6/J (WT) mice (8–12 weeks old) were purchased from the Jackson ImmunoResearch Laboratories (Bar Harbor, ME) and bred in-house. Mice, both male and female, were randomly allocated to experimental groups of ≥ 4 mice per group. All experiments were performed with age- and sex-matched mice housed in a pathogen-free barrier facility. All protocols were in agreeance with the guidelines established by the Institute of Animal Care and Use Committee at the Albert Einstein College of Medicine in compliance with the National Institutes of Health’s Guide for Care and Use of Laboratory Animals.

### Induction of myelin oligodendrocyte glycoprotein-induced experimental autoimmune encephalomyelitis

To induce EAE, all mice were immunized with MOG_35–55_ peptide (MEVGWYRSPFSRVVHLYRNGK; CelTek Bioscience, Nashville, TN) emulsified in an equal volume of complete Freund’s adjuvant (CFA; BD Difco, Franklin Lakes, NJ) and pertussis toxin (Ptx; List Biological Laboratories, Campbell, CA). Mice were anesthetized with isoflurane, and MOG_35–55_:CFA (100 μL; 3 mg/mL) was subcutaneously injected on each hind flank (200 μL total/mouse) on day 0. Ptx (200 μL; 2.5 μg/mL) was administered intraperitoneally (IP) on day 0 and day 2 post-MOG immunization (dpm). Mice were monitored daily for clinical symptoms and were graded as follows: 0 = no clinical symptoms, 1 = flaccid tail, 2 = flaccid tail and hind limb weakness, 3 = hind limb paralysis, 4 = hind limb paralysis and forelimb weakness, and 5 = moribund. Mice that did not present with clinical symptoms were excluded from the analysis (~ 5% of total). Acute EAE was defined as < 10 days with clinical symptoms of EAE and chronic phases as > 15 days with clinical symptoms (typically > 20 dpm).

### Administration of activating antibodies

Mice were injected IP with α-Axl (Antigen Affinity-purified Polyclonal Goat IgG (AF854); R&D Systems, Minneapolis, MN), α-Mer (AF591; R&D Systems), control IgG (AB-108-C: R&D Systems), or 1× PBS. The α-Axl and α-Mer antibodies are known to activate their respective receptors [28]. Dosing were as follows: (1) 10 μg every other day for a total of 40 μg starting at 8 dpm (preclinical), (2) 10 μg every other day for a total of 40 μg starting at 12 dpm, (3) 40 μg at day 8 dpm, and (4) 40 μg every other day for a total of 80 μg starting at 8 dpm. We observed the greatest effect with dose 3, 40 μg at day 8 dpm, and that dose was selected for all subsequent experiments. Mice were sacrificed at varying time points post-MOG immunization, and tissues were analyzed fresh, snap frozen, or fixed in 4% paraformaldehyde (PFA) for further analysis.

### Immunohistochemistry

Mice were anesthetized with isoflurane and sacrificed by total body perfusion with 4% PFA. The spinal cords were excised and placed in 4% PFA fixative and paraffin-embedded for immunohistochemistry (IHC). Paraffin-embedded sections of the lumbar region of the spinal cord were immersed in xylene and rehydrated with descending concentrations of ethanol and 1× Tris-buffered saline (TBS), pH = 7.4. Antigen retrieval was performed by microwaving slides in distilled boiling water or 1 mM EDTA (CD3 and FoxP3) for 14 min. The sections were then incubated in 1× TBS containing 0.25% Triton X-100 and 3% hydrogen peroxide for 20 min at 25 °C. Blocking was achieved by incubating the sections in 5% non-fat milk and 5% goat serum for 1 h at 25 °C.

The sections were incubated with the primary antibodies in 5% non-fat milk at the specified dilutions: monoclonal antibody (mAb) to myelin basic protein (MBP) (SMI99; 1:1000; EMD Millipore, Danvers, MA), mAb neurofilament H non-phosphorylated (SMI32; 1:20,000; EMD Millipore), mAb α-CD3 (1:150; Agilent Dako, Santa Clara, CA), polyclonal antibody (pAb) α-Iba1 (1:400; Agilent Dako), and α-FoxP3 (1:150; eBioscience Inc., San Diego, CA). The sections were then washed 3× with 1× TBST and subsequently incubated with the appropriate corresponding secondary antibodies followed by detection with the species-appropriate Vecta Staining Kit (Vector Laboratories) and visualized by diaminobenzidine (DAB; Sigma). For immunofluorescent staining, fluorophore-conjugated secondary antibodies were used followed by staining with 4,6-diamidino-2-phenylindole (DAPI, 1:1000; Thermo Fisher). The sections were mounted using aqueous mounting media (ProlongGold, Invitrogen) and visualized using fluorescent microscopy at the indicated magnifications or scanned using Pannoramic 250 Flash III Slide Scanner (3DHistech, Budapest, Hungary).

Histological sections were graded on a scale of 0–4. For Iba1^+^ and CD3^+^ staining, the coronal sections of the spinal cords or brains were scored on a 0–4 inflammatory scale where a score of 0 is the equivalent pathology observed in a naive mouse, 1 = mild inflammation, 2 = moderate, 3 = severe inflammation, and 4 = very severe inflammation involving 50% or more of the tissue [29]. For relative demyelination after MBP staining, the scores were assigned as follows: 0 = MBP immunoreactivity observed in naive mice, 1 = mild demyelination, 2 = moderate demyelination, 3 = severe demyelination, and 4 = very severe involving > 50% of white matter.

For relative axonal damage, we quantified the SMI32^+^ axonal swellings in both the left and the right ventral regions of the white matter of mouse spinal cords using multiple 20× fields: 0 = 0 SMI32^+^ axonal swellings as observed in naive mice, 1 = ≤ 10 SMI32^+^ swellings, 2 = 10–20 SMI32^+^ swellings, 3 = 20–50 SMI32^+^, and 4 = ≥ 50 SMI32^+^ swellings. Slides were blinded, and at least three sections of the lumbar spinal cord for each animal were assessed by two individuals; the number of animals used for each experiment is indicated in the figure legends.

### ELISA

Protein homogenates were prepared from freshly isolated spinal cords and whole brains and mechanically homogenized in 1 mL of 1× PBS containing protease inhibitors (Pierce Protease Inhibitor Tablets, Thermo Fisher Scientific, Grand Island, NY). Total protein content was measured using a Pierce BCA Protein Assay kit (Thermo Fisher Scientific), following the manufacturer’s instructions. Homogenates were subsequently centrifuged at 20,000×*g* for 20 min at 4 °C. Supernatants were collected and diluted, and 100 μL was loaded into sandwich ELISA plates following the manufacturer’s protocol. Mouse IL-1β, IL-6, IL-17, IFN-γ, IFN-β, and TNF-α DuoSet ELISA kits (R&D Systems) were used. The RayBio Mouse Axl ELISA kit was used to detected protein levels of Axl following the manufacturer’s protocol (RayBiotech, Norcross, GA).

### qRT-PCR

RNA was extracted from the brain and spinal tissue using TRIzol reagent (Thermo Fisher Scientific). For the brain sections, RNA was extracted from a 2-mm section of the corpora callosa. Total RNA was quantified using a NanoDrop ND-2000 spectrophotometer (Thermo Fisher) at an absorbance ratio of 260 and 280 nm, and 1 μg RNA was used for cDNA synthesis. Reverse transcription was carried out using the iScript cDNA Synthesis Kit (Bio-Rad, Hercules, CA). For qRT-PCR reactions, templates were diluted 1:3 in a PCR mix containing each gene-specific primer pair and iTaq Universal SYBR Green Supermix (Bio-Rad). Gene expression was measured on a StepOne Plus Real-Time PCR system (Applied Biosystems). All samples were run in triplicate and normalized to the geometric mean of HPRT or GAPDH with a WT sample as a reference. Melting curves were analyzed for each sample to confirm the specificity of the amplicon. Fold change was determined using the 2^−ΔΔCt^ method. The following primers were used: TNF-α—forward - TGTAGCCCACGTCGTAGCAA, reverse - AGGTACAACCCATCGGCTGG; IFN- γ—forward - AAAGAGATAATCTGGCTCTGC, reverse - GCTCTGAGCAATGAACGT; IL-1β—forward - TGTGCAAGTGTCTGAAGCAGC, reverse - TGGAAGCAGCCCTTCATCTT; IL-2—forward - CCCAAGCAGGCCACAGAATTGAAA, reverse - TGAGTCAAATCCAGAACATGCCGC; IL-4—forward - GGTCTCAACCCCCAGCTAGT, reverse - GCCCGATGATCTCTCTCAAGTGAT; IL-6—forward - ATTGGATGCTTACCAAACTGGAT, reverse - TGAAGGACTCTGGCTTTGTCT; IL-10—forward - GCTCTTACTGACTGGCATGAG, reverse - CGCAGCTCTAGGAGCATGTG; SOCS1—forward - CTGCGGCTTCTATTGGGGAC, reverse - AAAAGGCAGTCGAAGGTCTCG; SOCS3—forward - ATGGTCACCCACAGCAAGTTT, reverse -TCCAGTAGAATCCGCTCTCCT.

### Isolation of CNS cells

Single-cell suspensions were prepared from the brain and spinal cord isolated from WT mice. CNS tissue was dissociated into single cells using the Neural Tissue Dissociation Kit (T) (Miltenyi, Auburn, CA), following the manufacturer’s instructions with adaptations. A 25% Percoll density gradient medium was used to remove any contaminating myelin.

### FACS analysis

To evaluate populations of immune cell subsets, single-cell suspensions were prepared and flow cytometry performed. Mice having consecutive clinical scores for 5–7 days were perfused with cold PBS, and the spinal cords and brains isolated and prepared for flow cytometry. Cells were blocked with mouse α-CD16/32 and surface stained with α-CD4 and α-CD25, α-CD11c, α-CD45, and α-CD127. Samples were acquired on an LSR II flow cytometer using the FACSDiva software, and analysis was performed using the FlowJo software.

### Statistical analysis

Statistical analyses were performed using the GraphPad Prism software (Prism Software, Lake Forest, CA). For parametric analysis, Student’s *t* test was used, and for non-parametric analysis, the Mann-Whitney *U* test and 2-way ANOVA were used. Statistical significance is presented as *p* ≤ 0.05.

## Results

### An activating α-Axl antibody significantly reduces the clinical course of MOG-induced EAE

**Fig. 1:**
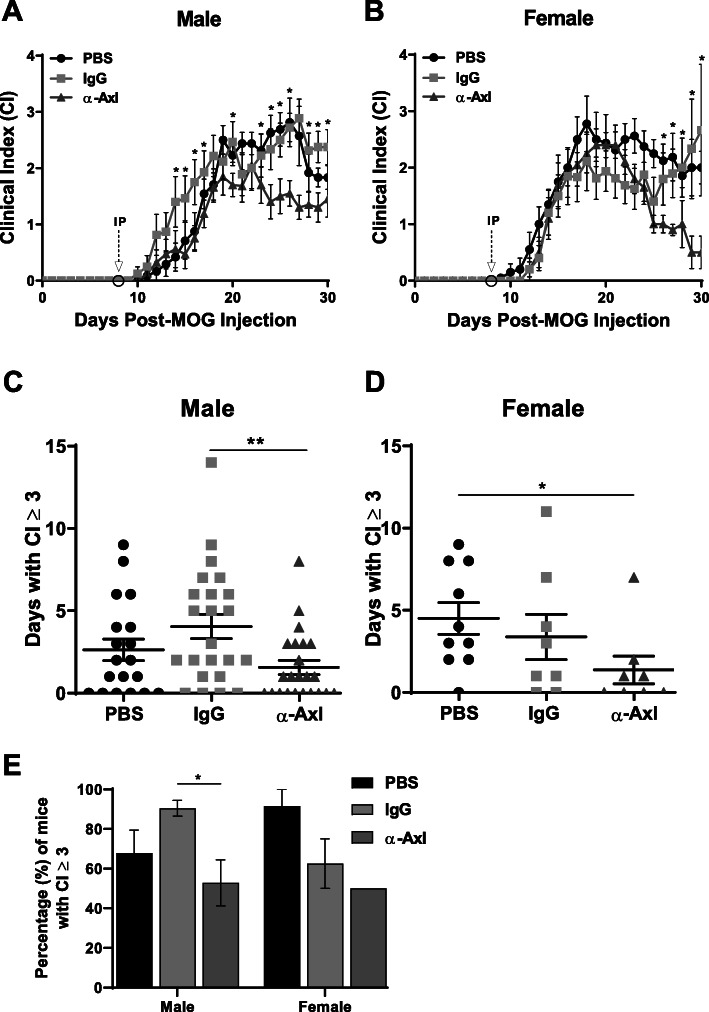
α-Axl antibody significantly reduces the clinical course and severity of MOG-induced EAE. C57Bl/6J mice were injected intraperitoneally (IP) with PBS, IgG isotype control, or α-Axl-activating antibody at 8 days post-MOG immunization. Mice were monitored daily, and the clinical index (CI) was recorded (*n* = 13–17/group). The clinical course of male (**a**) and female (**b**) mice during acute and chronic EAE. The number of days male (**c**) and female (**d**) mice maintained a CI ≥ 3. **e** Percentage of mice with a CI ≥ 3. Statistical significance represents the comparison of IgG versus α-Axl-treated mice. *p ≤ 0.05 and **p ≤ 0.01, Mann-Whitney *U* test

We have previously demonstrated that with continual intracerebroventricular GAS6 delivery by a micro-osmotic pump at a set flow rate (Alzet #1104, 0.11 ul/h, 28 d), the activity of the Gas6 receptor resulted in a significant reduction in the severity of EAE symptoms [18]. Based on these observations, we predicted that mice treated with an activating α-Axl antibody would also have a less severe disease course. Prior to the onset of EAE symptoms (8 days post-MOG immunization; dpm), and a time point in which T cells have entered the CNS [30], three groups of mice were intraperitoneally (IP) injected with 40 μg of an activating α-Axl antibody, IgG isotype control, or PBS. Male mice injected with the activating α-Axl antibody had significantly lower clinical scores during both the acute (< 10 days with clinical symptoms) and chronic (> 15 days with clinical symptoms of EAE) phases of the disease compared to control mice (Fig. 1a). In female mice, significantly lower clinical scores were observed during the chronic EAE phase compared to the control groups (Fig. 1b). At 30 dpm, the average score of α-Axl antibody-treated male mice (*n* = 17) was 1.5 ± 0.3 versus 2.4 ± 0.3 for the IgG-treated mice (*n* = 16) (*p* = 0.02) and 1.8 ± 0.2 (*n* = 12) for the PBS-treated controls (Fig. 1a). In female mice, the average score of mice treated with the α-Axl antibody (*n* = 10) was 0.5 ± 0.3 versus 2.7 ± 1.2 for the IgG-treated mice (*n* = 10) (*p* = 0.02) and 2.0 ± 0.3 for the PBS-treated controls (*n* = 10) (Fig. 1b).

Axl antibody treatment also reduced the severity of EAE. Both female and male mice treated with the α-Axl antibody had significantly fewer days with a clinical score of ≥ 3 relative to the PBS-treated controls (Fig. 1c, d). In addition, only 47% of male mice treated with α-Axl obtained a clinical score of ≥ 3, compared to 83% and 69% of IgG- and PBS-treated controls, respectively (Fig. 1e). In female mice, 50% treated with α-Axl obtained a clinical score of ≥ 3, compared to 60% and 90% of IgG- and PBS-treated controls, respectively (Fig. 1e). As a result of the significant reduction in the severity of the EAE symptoms observed in male mice, this grouping was selected for subsequent analyses.

### α-Axl antibody treatment reduces demyelination during chronic EAE

**Fig. 2:**
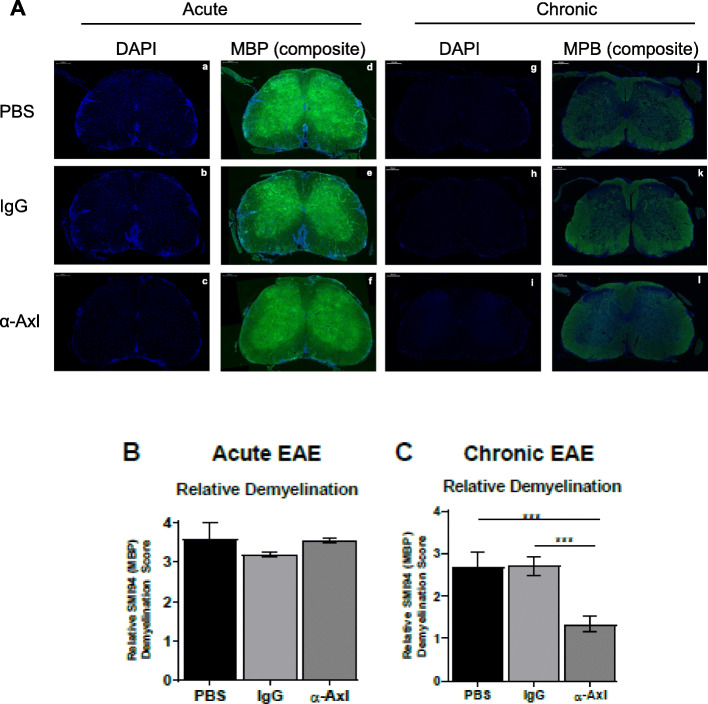
Demyelination is significantly reduced during chronic EAE in response to α-Axl antibody treatment. Representative lumbar spinal cord sections from C57Bl/6J mice treated with PBS (*n* = 6), IgG isotype control (*n* = 6), or α-Axl-activating antibody (*n* = 6) analyzed by IHC. **A** Immunofluorescent staining of the representative spinal cord sections during acute (a–f) and chronic (g–l) EAE. blue = DAPI, (d–f) composite of MBP (green) and DAPI. Quantification of MBP demyelination during acute (**B**) and chronic (**C**) EAE by scoring the criteria detailed in the “Methods” section. *p ≤ 0.05 and **p ≤ 0.01, Mann-Whitney *U* test. All scale bars are 200 μm

To determine whether Axl activation affects myelination and effectively reduces demyelination, we used IHC to quantify the levels of myelin in the spinal cords of treated mice during EAE, using the 0–4 demyelination score detailed in the “Methods” section. During acute EAE, we observed no quantifiable differences in MBP immunoreactivity in the lumbar spinal cord of α-Axl antibody-treated mice relative to IgG-treated mice (Fig. 2A (a–f), B). However, during chronic EAE, significantly more MBP^+^ cells were observed in the lumbar spinal cord of α-Axl antibody-treated mice compared to IgG- and PBS-treated mice (Fig. 2A (g–i)), reflected by the decrease in relative demyelination (Fig. 2C). Our data demonstrates that there was less demyelination in the spinal cords in response to α-Axl antibody treatment relative to controls, where larger areas of demyelination were visible in the ventral and lateral regions of the spinal cord.

### α-Axl antibody treatment reduces the number of Iba1^+^ microglia in the spinal cord during chronic EAE

**Fig. 3:**
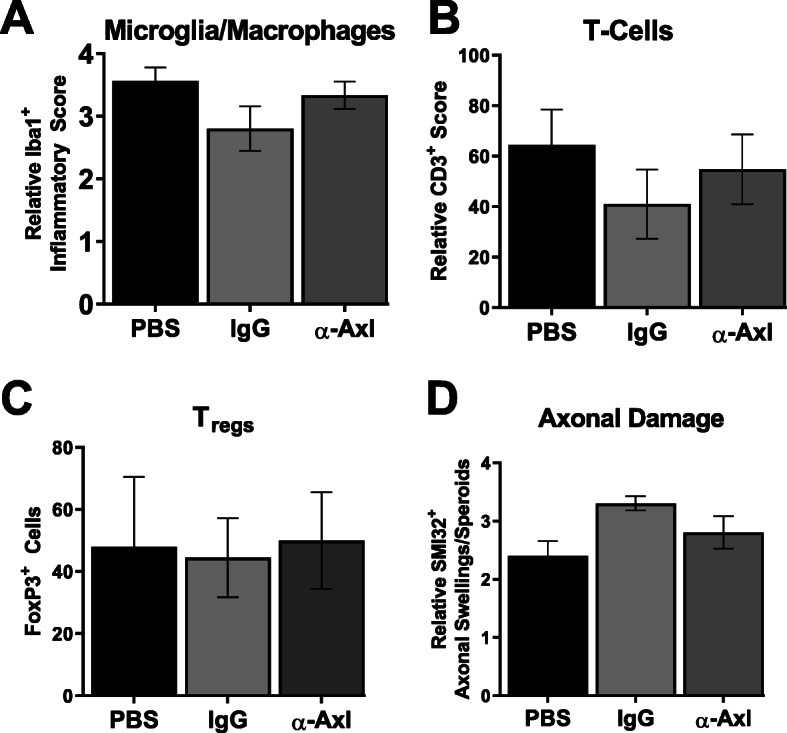
α-Axl antibody treatment has no effect on the number of Iba1^+^ microglia, T cells, T_regs_, or axonal damage in the spinal cord during acute EAE. Quantification of Iba1 (**a**), CD3 (**b**), FoxP3 (**c**), and SMI32 (**d**) immune-stained lumbar spinal cord sections of PBS- (*n* = 6), IgG isotype control- (*n* = 6), or α-Axl-activating antibody-treated (*n* = 6) mice during acute EAE. Quantification of the SMI32^+^ axonal swellings (> 3 μm) was conducted on multiple × 20 fields of the left and the right ventral regions of the lumbar spinal cord. *p ≤ 0.05, Mann-Whitney *U* test

**Fig. 4:**
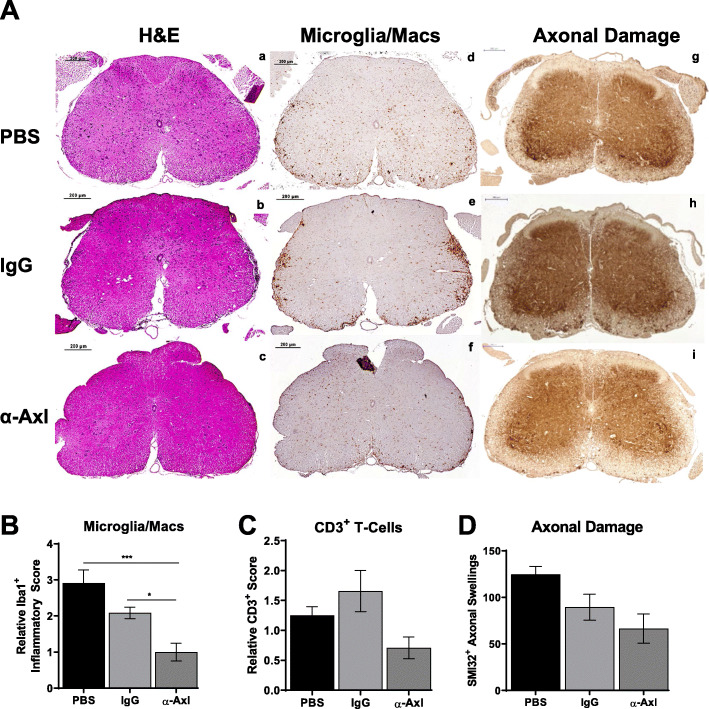
α-Axl antibody treatment significantly reduces the number of Iba1^+^ microglia in the spinal cord during chronic EAE. **A** Representative lumbar spinal cord sections from PBS- (*n* = 3), IgG isotype control- (*n* = 3), or α-Axl-activating antibody-treated (*n* = 4) mice during chronic EAE. (a–c) H&E, (d–f) Iba1^+^ microglia/macrophages, and (g–i) SMI32^+^ axonal swellings (scale bar = 100 μm). Quantification of **B** Iba1, **C** CD3, and **D** SMI32 axonal swellings. *p ≤ 0.05, **p ≤ 0.01, and ***p ≤ 0.001, Mann-Whitney *U* test

The lumbar region of the spinal cord was isolated during acute and chronic disease phases for pathologic analyses. H&E and immunostaining were used to determine whether there is a reduction in inflammatory cells (fewer activated Iba1^+^ cells and infiltrating CD3^+^ T cells) and fewer SMI32^+^ axonal spheroids and dystrophic axons indicative of axonal damage. Treatment with the α-Axl antibody had no effect on the number of inflammatory Iba1^+^ cells, infiltrating CD3^+^ T cells, or FoxP3^+^ regulatory T cells (T_regs_) in the spinal cord during acute EAE (Fig. 3a–c). In addition, no significant differences in the number of SMI32^+^ axonal spheroids were observed during the acute phase of the disease (Fig. 3d). Conversely, α-Axl antibody treatment significantly reduced the number of Iba1^+^-activated microglia/macrophages in the lumbar spinal cord during chronic EAE (Fig. 4A (d–f), B), and although the number of infiltrating CD3^+^ T cells or SMI32^+^ axonal swellings/spheroids decrease, significance was not achieved (Fig. 4A (g–i), C, D).

**Fig. 5:**
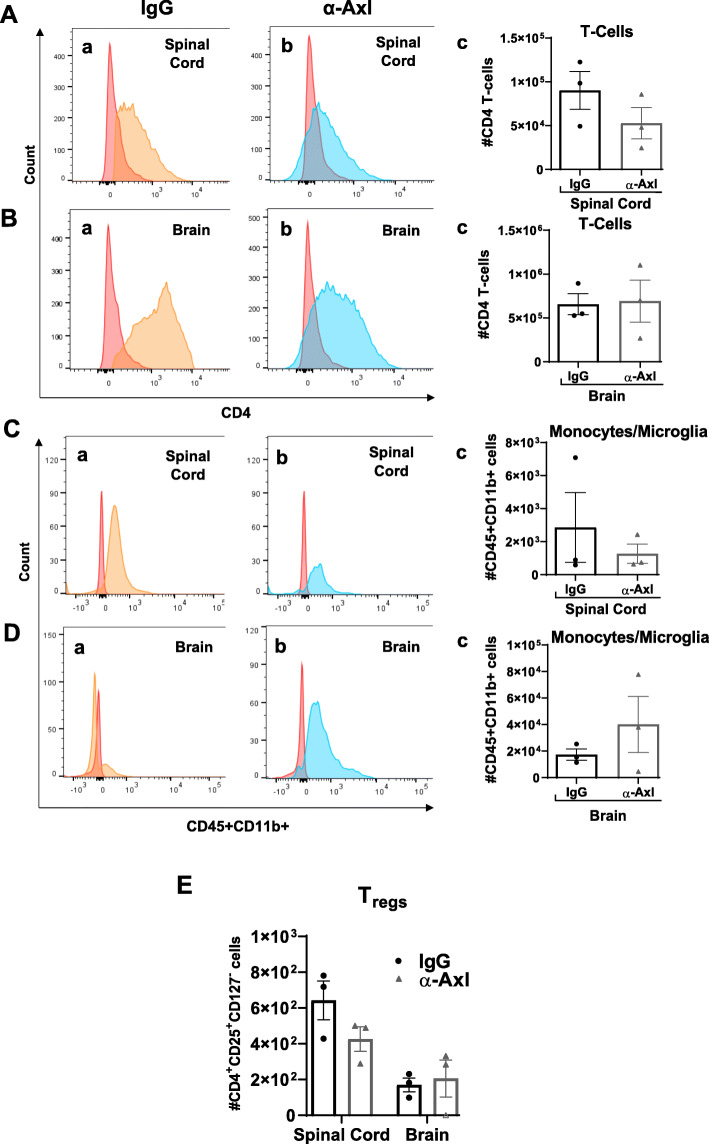
Populations of T cells, monocytes/microglia, and T_regs_ in the spinal cord and brain of mice treated with α-Axl remained unchanged compared to IgG control mice during chronic EAE. Flow cytometric analysis of the total CD4^+^ T cells in the **A** spinal cord and **B** brain and total CD45^+^CD11b^+^ monocytes/microglia in the **C** spinal cord and **D** brain. **E** Quantification of CD4^+^CD25^+^CD127^−^ T_regs_ in the brain and spinal cord of IgG isotype control and α-Axl mice during chronic EAE (*n* = 3/ group; analyses were performed using Mann-Whitney *U* test and one-way ANOVA)

The significant decrease in Iba1^+^-activated microglia/macrophages in response to α-Axl antibody treatment led us to examine the populations of potentially inflammatory immune cells by flow cytometry. Brain and spinal cord tissues were isolated from IgG- and α-Axl-treated mice during chronic EAE (*n* = 3/group), when significant differences in the clinical indices were observed between the two groups of mice. We observed no significant differences in the populations of CD4^+^ T cells, CD45^+^CD11b^+^ monocyte-derived macrophages/microglia, or CD4^+^CD25^+^CD127^−^ T_regs_ in the spinal cords and brains of mice treated with α-Axl compared to IgG control mice (Fig. 5).

### Antibody activation of Axl does not affect the production of inflammatory cytokines or Axl protein in the spinal cord and brain during chronic EAE

**Fig. 6:**
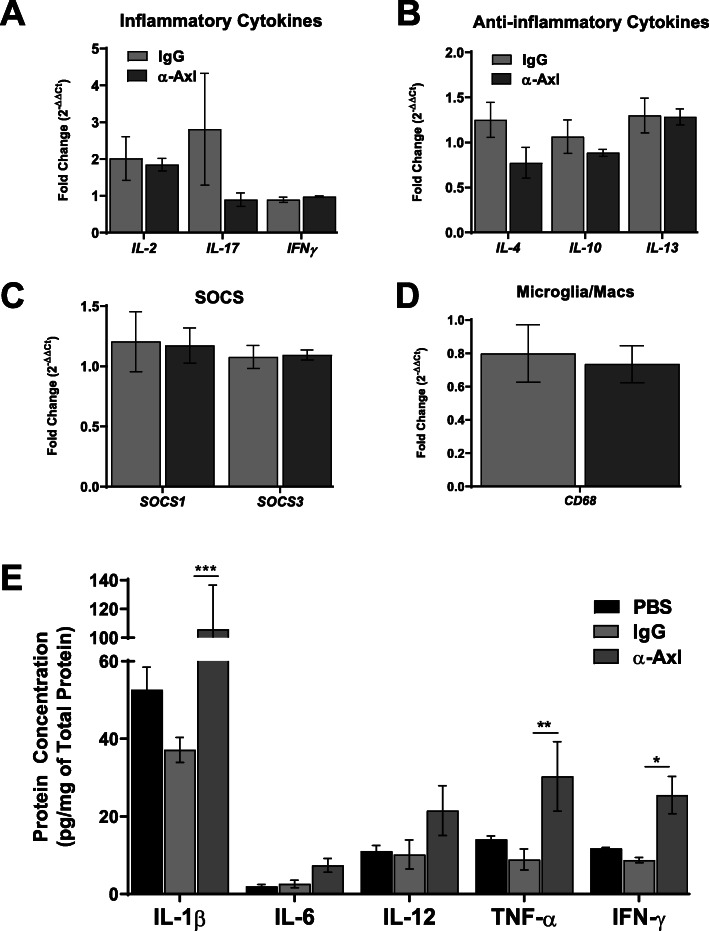
Axl activation does not affect the mRNA expression of inflammatory cytokines in the spinal cord but increases the production of IL-1β, TNF-α, and IFN-γ in the corpus callosum during acute EAE. RNA was extracted from the spinal cords of IgG isotype control- (*n* = 8) and α-Axl-treated (*n* = 7) mice during acute EAE. **a** mRNA expression of pro-inflammatory cytokines IL-2, IL-17, and IFN-γ; **b** anti-inflammatory cytokines IL-4, IL-10, and IL-13; **c** suppressors of cytokine signaling SOCS1 and SOCS3; and **d** microglia/macrophage marker CD68 during acute EAE. All genes were normalized to HPRT expression. Differences in mRNA expression are shown as 2^−ΔΔCt^. **e** Protein homogenates were prepared from the corpus callosum of IgG isotype control-, α-Axl-, and PBS-treated mice, and protein levels of IL-1β, IL-6, IL-12, TNF-α, and IFN-γ were measured by ELISA (pg/mg total protein) (*n* = 3–4 per group). Data were analyzed using 2-way ANOVA. *p < 0.05, **p ≤ 0.01, and ***p ≤ 0.001 are considered statistically significant

We examined the expression and production of inflammatory molecules during the peak of disease (5–7 days with EAE symptoms), because this is when the largest number of infiltrating immune cells are present in the CNS. Pro-inflammatory cytokines and chemokines are expressed in and secreted by multiple cells in the CNS, including astrocytes, microglia, infiltrating T cells, macrophages, and dendritic cells. RNA and protein were isolated from the spinal cord of mice having clinical scores (CI ≥ 1) for 5–7 consecutive days. No differences in the mRNA expression of pro-inflammatory cytokines IL-2, IL-17, or IFN-γ or anti-inflammatory cytokines IL-4, IL-10, or IL-13 were observed compared to IgG-treated mice (Fig. 6a, b). In addition, the mRNA expression levels of suppressors of cytokine signaling (SOCS) 1 and 3, and macrophage/microglia marker CD68 remained comparable (Fig. 6c, d).

**Fig. 7:**
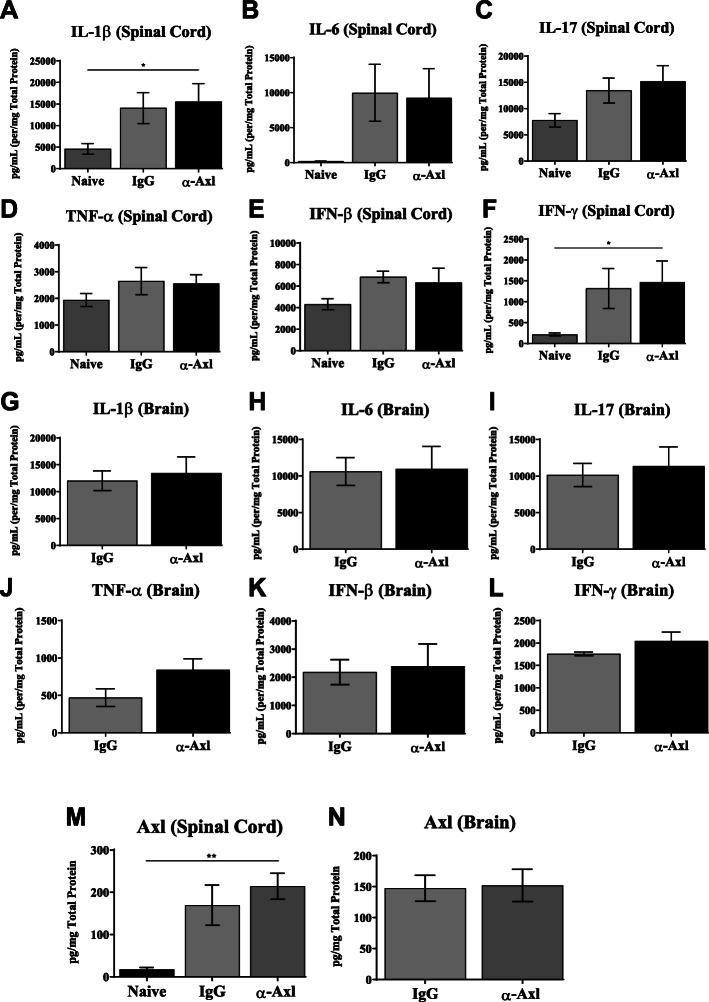
Axl antibody activation does not affect the production of inflammatory cytokines or Axl in the spinal cord and brain during chronic EAE. Protein was extracted from whole spinal cords and brains of chronically ill mice. Protein homogenates were prepared from naive, IgG isotype control, and α-Axl-treated mice, and protein levels of the spinal cord **a** IL-1β, **b** IL-6, **c** IL-17, **d** TNF-α, **e** IFN-β, and **f** IFN-γ were measured by ELISA (pg/mg total protein) (*n* = 8–11). Protein levels of **g** IL-1β, **h** IL-6, **i** IL-17, **j** TNF-α, **k** IFN-β, and **l** IFN-γ in the whole brain tissue were measured by ELISA (pg/mg total protein) (*n* = 8–11 per group). Protein levels of Axl in the **m** spinal cord and **n** brain were measured by ELISA (pg/mg total protein) (*n* = 6). Data were analyzed using 2-way ANOVA. *p < 0.05 and **p ≤ 0.01 are considered statistically significant

We examined the levels of specific cytokines in the corpus callosum during acute EAE (Fig. 6e) and in the whole brain and spinal cord during chronic EAE (Fig. 7) by ELISA. Although no differences were observed in IL-6 and IL-12 protein levels, IFN-γ, TNF-α, and IL-1β protein levels significantly increased in the corpus callosum during peak acute disease in response to α-Axl treatment (Fig. 6e). During chronic EAE, no differences were observed in the levels of IL-1β, IL-6, IL-17, TNF-α, IFN-β, and IFN-γ in the brain and spinal cord (Fig. 7a–l). However, it is important to note that IL-1β and IFN-γ protein levels significantly increased in the spinal cords of both groups of mice compared to naive mice (Fig. 7a–f).

We had previously shown that *Axl* mRNA expression is upregulated during EAE [22]; therefore, we measured the protein levels of Axl in the spinal cord and brain homogenates of α-Axl antibody-treated mice and compared it to that of IgG-treated mice during chronic EAE. We did not observe a difference in the levels of Axl protein between the two groups of mice; however, consistent with the previous findings, Axl production increased significantly in the spinal cord of α-Axl-treated mice compared to naive mice (7 M), while no differences were observed in the brain (7N).

### α-MerTK treatment has no effect on EAE outcome

**Fig. 8:**
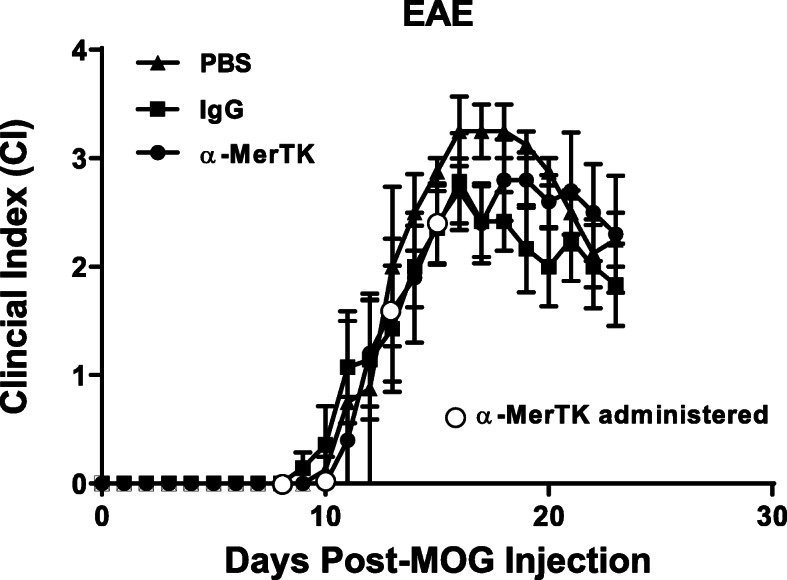
MOG-induced EAE clinical course in mice treated with an activating α-MerTK antibody. Male C57Bl/6J mice were injected intraperitoneally (IP) with PBS, IgG isotype control, or 10 μg of α-MerTK-activating antibody at 8, 10, 13, and 15 days post-MOG immunization (*n* = 7/group). Mice were monitored daily, and the clinical index (CI) was recorded. No significant differences were observed between the groups

As with α-Axl antibody-treated mice, groups of male and female mice were injected IP with 40 μg α-MerTK before the onset of EAE symptoms (8 dpm). We observed no differences in the EAE disease course or severity in response to α-MerTK treatment (Fig. 8).

## Discussion

Defects in TAM signaling disrupt multiple cellular processes that regulate homeostasis, phagocytosis, and inflammation [31–34]. The role of Gas6 in the regulation of inflammation and maintenance of homeostasis has been well established, and we have demonstrated that Gas6 enhances oligodendrocyte survival and myelination in vitro and in vivo [17, 35–38]. Axl is activated by the ligand-driven dimerization of Axl with Gas6 [39]. Our studies have shown that *Axl* expression is upregulated during EAE, that Axl^−/−^ mice have a more severe clinical disease course and EAE pathology relative to WT mice, and that Tyro3^−/−^ mice have increased *Axl* expression [22, 24, 25]. Further, there is no difference in the clinical course of Tyro3^−/−^ and WT mice during EAE. Zagorska et al. also found that affinity-purified, polyclonal α-Axl (AF854; R&D Systems) and α-Mer (AF591; R&D Systems) activated their respective receptors in vitro and in vivo. Based on these studies, we predicted that the protective effects observed in response to Gas6 treatment were due to Axl activation and that Gas6/Axl signaling functions in the regulation and maintenance of axonal integrity and myelination in the CNS.

Our previous study showed that the delivery of GAS6 influences oligodendrocyte progenitor cell maturation, enhances myelination, and preserves axonal integrity. Therefore, we examined whether a single α-Axl treatment during EAE would be beneficial for myelin preservation. We injected mice with an affinity-purified, polyclonal α-Axl antibody that has been shown to activate only the Axl receptor and does not activate MerTK or Tyro3 [28]. In addition, the α-MerTK antibody has been shown to only activate MerTK, and IgG does not activate the TAMs or Gas6 in vivo [28]*.* When treated before the onset of EAE symptoms (8 dpm), a period after peripheral inflammatory cells have populated the CNS, we found that Axl receptor activation consistently reduced the severity of EAE symptoms, and α-Axl antibody-treated mice maintained clinical indices below that of both PBS- and IgG-treated controls during the chronic phase of the disease. In addition, the α-Axl antibody-treated mice had fewer days paralyzed (Fig. 1). We also evaluated the effect of Axl activation with increased concentrations of α-Axl (80 μg), and after the onset of EAE symptoms but before peak disease (10–16 dpm), and observed no significant differences in the disease course or pathology (Supplemental Fig 1). In fact, increasing the dose of the α-Axl from 40 to 80 μg did not enhance the benefits of Axl activation.

Our study demonstrates that IP injection of an α-Axl antibody significantly reduces demyelination during chronic EAE (Fig. 2). In addition, there was a decrease of ~ 30% in the number of SMI32^+^ axonal swellings/spheroids, an indicator of axonal damage in the α-Axl antibody-treated group during chronic EAE relative to the IgG-treated group; however, statistical significance was not achieved (Fig. 4). The significant reduction in the degree of demyelination, measured by the increase in MBP^+^ immunoreactive cells in the spinal cord during chronic EAE, is suggestive of a protected myelin sheath. These results directly contrast with what we have observed in Axl^−/−^ mice during EAE, where increased loss of myelin, increased accumulation of myelin debris, and significantly more SMI32^+^ axons were observed [24]. This study also demonstrates that α-Axl antibody treatment significantly reduces the number of Iba1^+^-activated microglia/macrophages in the spinal cords of mice with clinical scores for ≥ 10 days, a period when α-Axl antibody-treated mice had lower clinical scores compared to control. Although not significant, we also observed a trend towards decreased CD3^+^ T cells and CD4^+^ T cells in the spinal cords of α-Axl-treated mice by IHC and FACS, respectively (Fig. 4).

During chronic EAE, the majority of Iba1^+^ cells in the CNS are microglia, and in analyzing the distribution of monocytes/macrophages (CD45^+^CD11b^+^) in the CNS by FACS, these cells were unchanged between the groups. However, due to the small number of inflammatory cells present during chronic EAE, we were unable to obtain detectable numbers of CD11b^+^CD45^med^ microglia in the CNS in both groups of mice by FACS.

To further examine the role of Axl in maintaining the integrity of the CNS, we analyzed select cytokines in the corpus callosum, brain, and spinal cord during acute and chronic EAE. During acute EAE, a period when activated inflammatory cells infiltrate the CNS and contribute to glial activation, we observed a significant increase in the production of IFN-γ, TNF-α, and IL-1β specifically in the corpus callosum of α-Axl antibody-treated mice relative to control. The mRNA expression of *IL-2*, *IL-17*, *IFN-γ*, *IL-4*, *IL-10*, *IL-13*, *SOCS1/3*, and *CD68* was similar between the α-Axl- and IgG control-treated mice. It is possible that the isolated corpus callosum included border-associated macrophages and T cells that did not invade the brain parenchyma, and we are examining that possibility in future studies. While it is possible that effects of Axl activation via an α-Axl antibody may differ by cell type or region, the increase in cytokine expression in the corpus callosum did not appear to affect the integrity of the spinal cord, where disease severity begins in the sacral region and travels up the cord. At the time of increased cytokine expression in the corpus callosum, there were no differences in axon or myelin integrity in the spinal cord. During chronic EAE, the protein levels of the inflammatory cytokines IL-1β, IL-6, IL-17, TNF-α, IFN-β, and IFN-γ significantly increased in the spinal cord of all mice relative to naive mice, consistent with the severity of the disease. However, no significant differences in the production of these cytokines were observed in the whole brain and spinal cord isolated from α-Axl- and IgG-treated mice. While the severity of the disease is mediated at least in part by the regulation of pro-inflammatory cytokines, we postulate that Axl activation in resident cells of the CNS may have a more profound effect on protecting the neurons, oligodendrocytes, and myelin sheath from the seemly dangerous cytokine milieu.

Similar to GAS6 treatment, which significantly decreased the clinical scores of mice relative to the control mice throughout EAE, we observed a significant reduction in the clinical indices of male mice treated with the α-Axl antibody [22]. Gas6 treatment did not affect the number of Iba1^+^-activated macrophages/microglia relative to control-treated mice; however, the number of SMI32^+^ swelling/spheroids significantly decreased with less demyelination in response to Gas6 treatment [22]. Therefore, both Gas6 treatment directly into the brain and a single anti-Axl antibody injection administered IP resulted in the protection from myelin loss during chronic EAE.

## Conclusions

The Gas6/TAM signaling pathway is known to regulate the innate immune response and enhance phagocytosis in macrophages/microglia [40, 41]. Gas6 is upregulated during acute EAE, and Gas6/TAM signaling has been shown to stimulate the generation of oligodendrocytes and increase myelin production in the adult CNS, including repair after demyelinating injury [18, 20]. In the CNS, Axl is widely expressed in microglia, oligodendrocytes, astrocytes, and neurons, and Gas6 is expressed and secreted by neurons. Gas6/Axl signaling is also mediated via autocrine and paracrine mechanisms. Therefore, with an improvement in EAE severity including fewer days with paralysis, less activated microglia/macrophages, and reduced demyelination in response to α-Axl antibody treatment, it is possible that other cell types may be contributing to Axl-induced protection in the absence of exogenous Gas6 treatment. In summary, our data suggests that intraperitoneal injection of an activating α-Axl antibody, but not an α-MerTK antibody, can have beneficial effects including lowering the severity of disease during chronic EAE.

## Supplementary information

**Additional file 1: Supplemental Figure 1.** EAE clinical course of mice treated with different concentrations of α-Axl antibody at varying time points. Male C57Bl/6J mice were injected **(A)** intraperitoneally (IP) with PBS, IgG isotype control (10 μg), or α-Axl activating antibody (10 μg), each at days 10, 12, 14, and 16 post MOG-immunization. **(B)** Male C57Bl/6J mice injected (IP) with PBS, IgG isotype control (40 μg), or α-Axl activating antibody (40 μg) each at days 10 and 12 MOG-immunization. Clinical course of female **(C)** and male **(D)** mice injected intravenously (IV) with PBS, IgG isotype control (10 μg), or α-Axl activating antibody (10 μg) every other day for a total of 40 μg starting at day 13 (female) or 14 (male) post MOG-immunization. Statistical significance represents comparison of IgG vs. α-Axl treated mice. *p ≤ 0.05; Mann-Whitney *U-*test.

## Data Availability

All data generated or analyzed during this study are included in this published article.

## Abbreviations

C57Bl6/J: WT mouse strain used for MOG-induce EAE
CI: Clinical index
CNS: Central nervous system
DPM: Day post-MOG immunization
EAE: Experimental autoimmune encephalomyelitis
Foxp3: Forkhead box protein 3
Gas6: Growth arrest-specific gene 6
H&E: Hematoxylin and eosin stain
Iba1: Ionized calcium adaptor-binding protein-1
IFN-γ: Interferon gamma
IHC: Immunohistochemistry
IL: Interleukin
IP: Intraperitoneal
MBP: Myelin basic protein
MOG: Myelin oligodendrocyte glycoprotein
MS: Multiple sclerosis
Pros1: Protein S
Ptx: Pertussis toxin
qRT-PCR: Quantitative real-time polymerase chain reaction
TAM: Tyro3, Axl, and MerTK
TBS: Tris-buffered saline
TBST: Tris-buffered saline with 0.1% Tween20
TNF-α: Tumor necrosis factor alpha
WT: Wild-type
