# Supplementary material for: Anti-Axl antibody treatment reduces the severity of experimental autoimmune encephalomyelitis
Source: J Neuroinflammation. 2020 Oct 29;17:324. doi: 10.1186/s12974-020-01982-3 (PMC7599105; doi:10.1186/s12974-020-01982-3)
Supplement: Supplementary file 1 — Additional file 1: Supplemental Figure 1. EAE clinical course of mice treated with different concentrations of α-Axl antibody at varying time points. Male C57Bl/6J mice were injected (A) intraperitoneally (IP) with PBS, IgG isotype control (10 μg), or α-Axl activating antibody (10 μg), each at days 10, 12, 14, and 16 post MOG-immunization. (B) Male C57Bl/6J mice injected (IP) with PBS, IgG isotype control (40 μg), or α-Axl activating antibody (40 μg) each at days 10 and 12 MOG-immunization. Clinical course of female (C) and male (D) mice injected intravenously (IV) with PBS, IgG isotype control (10 μg), or α-Axl activating antibody (10 μg) every other day for a total of 40 μg starting at day 13 (female) or 14 (male) post MOG-immunization. Statistical significance represents comparison of IgG vs. α-Axl treated mice. *p ≤ 0.05; Mann-Whitney U-test. [file 12974_2020_1982_MOESM1_ESM.pdf]

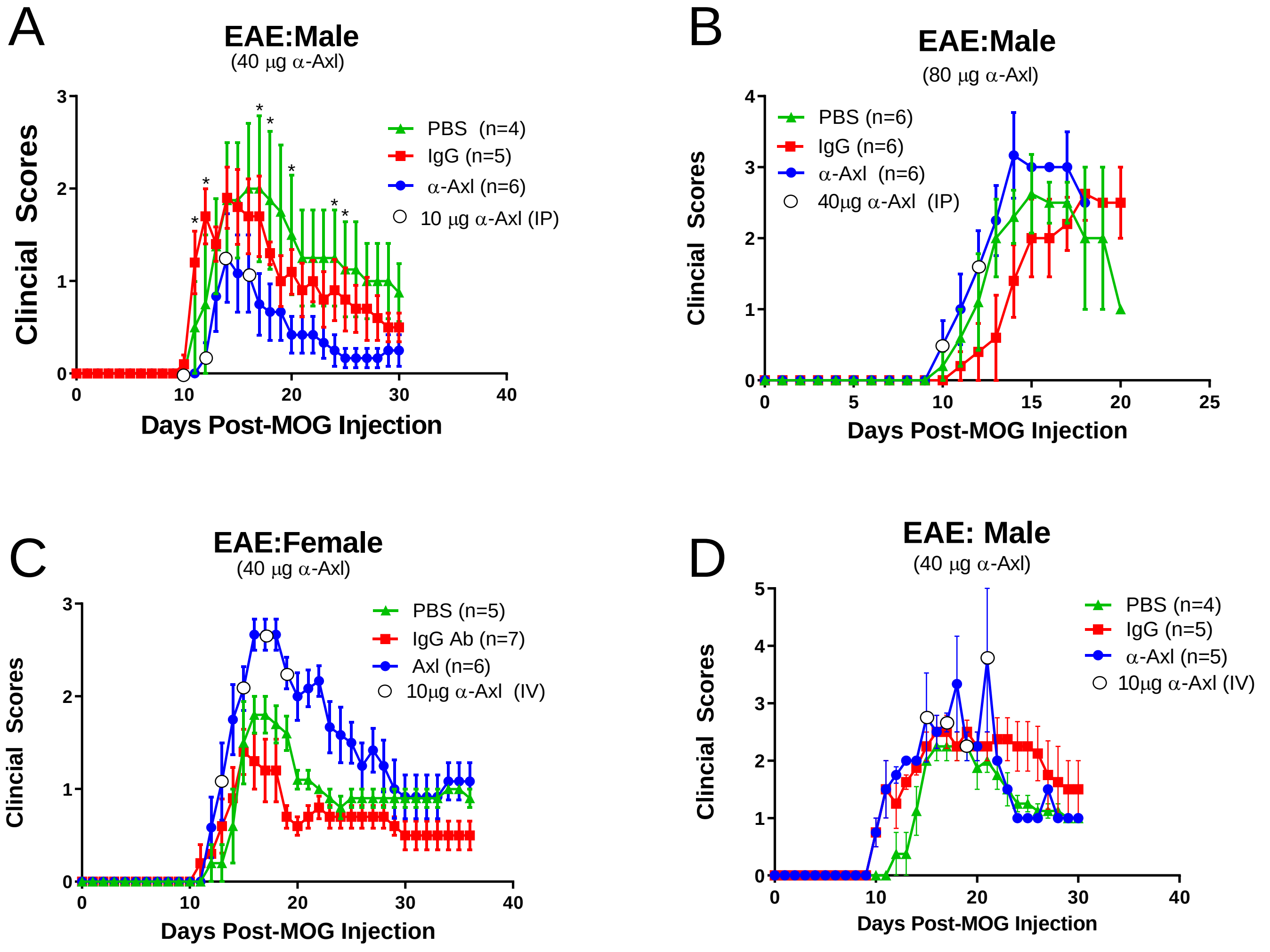

Supplemental Figure 1. EAE clinical course of mice treated with different concentrations of α-Axl antibody at varying time points. Male C57Bl/6J mice were injected **(A)** intraperitoneally (IP) with PBS, IgG isotype control (10 µg), or α-Axl activating antibody (10 µg), each at days 10, 12, 14, and 16 post MOG-immunization. **(B)** Male C57Bl/6J mice injected (IP) with PBS, IgG isotype control (40 µg), or α-Axl activating antibody (40 µg) each at days 10 and 12 MOG-immunization. Clinical course of female **(C)** and male **(D)** mice injected intravenously (IV) with PBS, IgG isotype control (10 µg), or α-Axl activating antibody (10 µg) every other day for a total of 40 µg starting at day 13 (female) or 14 (male) post MOG-immunization. Statistical significance represents comparison of IgG vs. α-Axl treated mice. *$p \leq 0.05$; Mann-Whitney *U*-test.
